# SUMO modification of LBD30 by SIZ1 regulates secondary cell wall formation in *Arabidopsis thaliana*

**DOI:** 10.1371/journal.pgen.1007928

**Published:** 2019-01-18

**Authors:** Chang Liu, Hasi Yu, Laigeng Li

**Affiliations:** National Key Laboratory of Plant Molecular Genetics and CAS Center for Excellence in Molecular Plant Sciences, Shanghai Institute of Plant Physiology and Ecology, Chinese Academy of Sciences, Shanghai, China; The University of North Carolina at Chapel Hill, UNITED STATES

## Abstract

A wide range of biological processes are regulated by sumoylation, a post-translational modification involving the conjugation of SUMO (Small Ubiquitin-Like Modifier) to protein. In *Arabidopsis thaliana*, *AtSIZ1* encodes a SUMO E3 ligase for SUMO modification. *siz1* mutants displayed defective secondary cell walls (SCWs) in inflorescence fiber cells. Such defects were caused by repression of *SND1/NST1*-mediated transcriptional networks. Yeast two-hybrid assay indicated that SIZ1 interacts with the LBD30 C-terminal domain, which was further confirmed using bimolecular fluorescence complementation and immunoprecipitation. Mass spectrometry and co-immunoprecipitation indicated that SIZ1 mediates SUMO conjugation to LBD30 at the K226 residue. Genes controlling SCW formation were activated by the overexpression of *LBD30*, but not in the *LBD30*_(*K226R*)_ mutant. *LBD30* enhancement of SCW formation resulted from upregulation of *SND1/NST1*-mediated transcriptional networks. This study presents a mechanism by which sumoylation of LBD30, mediated by SIZ1, regulates SCW formation in *A*. *thaliana*.

## Author summary

Secondary cell wall (SCW) is essential for upright plant growth and long-distance transport of water and solutes. Regulation of SCW formation at the transcriptional level has been much studied. Here we show that AtSIZ1, a small ubiquitin-related modifier (SUMO) E3 ligase, mediates the sumoylation of transcription factor LBD30, a process that regulates SCW formation. Elucidating a role of sumoylation in the post-translational regulation of SCW formation, complementing the body of research on regulation at the transcriptional level, provides additional insight into a key process that is essential for upright growth and the long-distance transport of water and solutes in plants.

## Introduction

Plant cells are surrounded by walls that provide structural support and regulate growth. All plant cells form primary cell walls, which are synthesized during cell expansion and differentiation, while specialized cell types can also deposit a secondary wall on the inside of the primary wall once cell elongation has finished. Examples of the SCW are found in vascular tissues, such as in fiber cells and tracheary elements, as well as in other mechanically important tissues, for example, collenchyma cells. The major constituents of the SCW are cellulose, non-cellulosic polysaccharides and lignin. These polymers are cross-linked, providing cell walls with both mechanical strength and hydrophobic properties. Such characteristics are needed for upright growth, long-distance transport of solutes[1], selectivity of nutrient and water transport in root endodermis[2], defense against pathogens[3], and phenomena such as pod shattering[4], anther dehiscence[5] and flower abscission [6].

In the cells undergoing SCW biosynthesis, SCW cellulose synthase complexes in the plasma membrane produce β-(1–4) glucan chains that assemble into microfibrils in the orientation guided by cortical microtubules [7]. The microfibrils are extruded into the cell wall matrix and interact with Golgi-synthesized hemicellulose, generally xylan and mannan, to form a stable network [8]. Lignin monomers are transported to the space within the polysaccharide network where they are oxidized and polymerized to make matured SCW [9]. Genes responsible for the SCW biosynthesis process are regulated by a group of transcriptional activators and repressors, which constitute a hierarchical regulatory network controlling SCW formation in various locations [10]. For example, *SND1* and *NST1* control SCW deposition in fiber cells [11–13] while *VND6* and *VND7* are responsible for vessel cells SCW formation in *A*.*thaliana* [14, 15]. Increasingly, post translational regulation of SCW formation is also being studied. For example, N-glycosylation regulates the enzyme activity of PtrMAN6 in suppression of SCW formation in *Populus* [16]. The phosphorylation of cellulose synthase AtCesA7 affected SCW cellulose biosynthesis in *A*.*thaliana* [17].

Sumoylation, conjugation of SUMO to substrate proteins, is a reversible and dynamic protein modification that regulates a range of biological processes [18]. SUMO conjugation forms a covalent bond between the C-terminal glycine carboxyl group of SUMO and the ε-amino group of a lysine residue, mostly occurring at the consensus motif ΨKXD/E (Ψ, hydrophobic amino acid; K, lysine for conjugation; X, any amino acid; D/E, acidic amino acids) of target proteins [19]. Completion of sumoylation requires an enzymatic cascade of SUMO E1 activating enzyme, SUMO E2 conjugating enzyme and SUMO E3 ligase[18]. This process can be reversed through desumoylating proteases [20]. Generally sumoylation results in either stabilization of the target protein by protecting it against ubiquitylation [21, 22] or destabilization by promoting the sumoylated protein for proteoasomal degradation[23]. Sumoylation can also alter protein cellular localization and modulate protein function or enzymatic activity[24]. In plants sumoylation plays a variety of roles in stress responses, growth, flowering, photomorphogenesis, nutrient homeostasis, and other biological processes[25, 26].

AtSIZ1 is an SP-RING (SIZ/PIAS-type) E3 ligase identified from *Arabidopsis thaliana*. It contains five structural domains including SAP (Scaffold attachment factor A/B//acinus/PIAS) domain, PINIT domain, SP-RING (SIZ/PIAS-RING) domain, SXS domain and PHD (Plant Homeodomain) [27]. These domains determine AtSIZ1 subcellular localization, enzyme activity, and action in responding to biotic and abiotic stresses [27]. AtSIZ1 plays various roles in growth [28], flowering[29, 30], light response[31, 32], immunity[33, 34] and metabolism of nutrient elements, such as phosphate[35], nitrogen [36] and copper[37]. AtSIZ1 is also implicated in sugar signaling [38]. Recent studies have shown that AtSIZ1 meidated sumoylation is involved in plant response to various stresses[26], including cold[39], heat stress[40], drought stress[41] as well as in signaling processes such as abscisic acid[42, 43], salicylic acid[44], auxin[45] and gibberellin signaling pathways[46].

In this study, we observed SCW defects in the *A*. *thaliana siz1* mutants. Genetic and biochemical analyses indicate that the SCW defects were caused by failure of the LBD30 sumoylation which was mediated by SIZ1. The study reveals a mechanism that sumoylation functions as a regulatory expedient in SCW formation in *A*. *thaliana*.

## Results

### *siz1* mutants display SCW defects in inflorescence fiber cells

**Fig 1:**
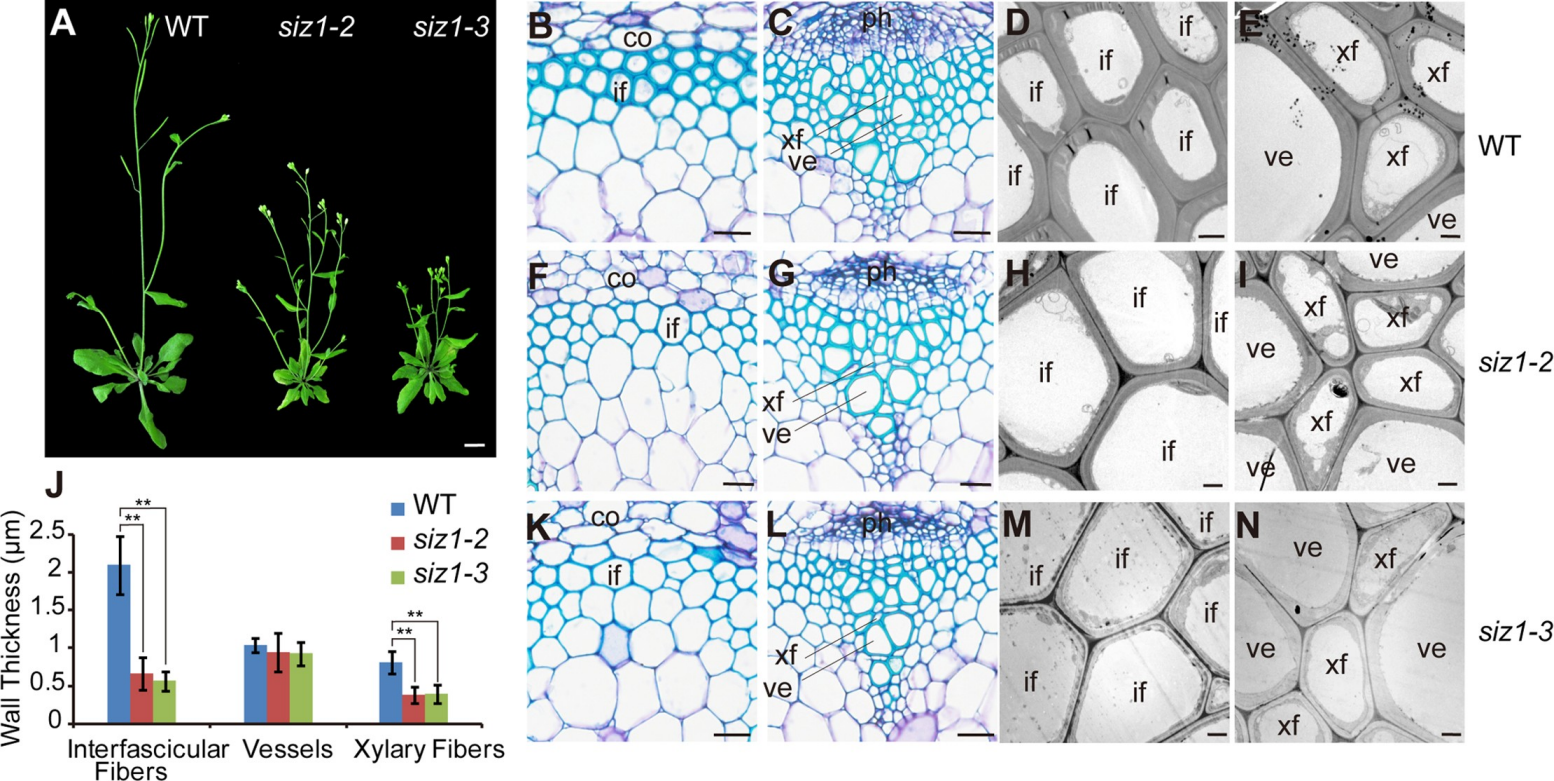
Inhibition of secondary cell wall thickening in *siz1* mutants. (A) Wild type (WT, Col-0), *siz1-2* and *siz1-3* mutant plants. (B), (F) and (K) Cross section of the interfascicular region of WT (B), *siz1-2* mutant (F) and *siz1-3* mutant (K) stems. (C), (G) and (L) Cross sections of the vascular bundle region of WT (C), *siz1-2* mutant (G) and *siz1-3* mutant (L) stems. (D), (H) and (M) Transmission electron micrographs of interfascicular fiber cells of WT (D), *siz1-2* mutant (H) and *siz1-3* mutant (M) plants. (E), (I) and (N) Transmission electron micrographs of xylem cells of WT (E), *siz1-2* mutant (I) and *siz1-3* mutant (N) plants. (J) Wall thickness of vessels and fibers in the inflorescence stems of WT and *siz1* mutants. Data represent average values±SD (n = 30 cells from 3 independent plants). **P < 0.01(Student`s *t*-test). co: cortex, if: interfascicular fiber, ph: phloem, ve: vessel, xf: xylary fiber. Scale bars = 10mm in (A), 20μm in (B), (C), (F), (G), (K) and (L), 2μm in (D), (E), (H), (I), (M) and (N). defects in SCWs. Specifically, interfascicular fiber cells and xylary fiber cells from the inflorescence stems of *siz1-2* (Fig 1F and 1G) and *siz1-3* (Fig 1K and 1L) showed a significant reduction in wall thickness compared to the wild-type (WT) (Fig 1B and 1C). Transmission electron microscopy analysis confirmed that *siz1* mutants form much thinner cell walls in the fiber cells (Fig 1D, 1E, 1H, 1I, 1M and 1N), while the wall thickness of vessel cells showed little difference between the *siz1* mutants and WT (Fig 1J).

We screened an *A*. *thaliana* T-DNA insertion pool (Col-0 background) for the phenotypic abnormality of SCW formation in the inflorescence stem through microscopy observation. Two T-DNA insertion alleles, *siz1-2* and *siz1*-*3*, which impair AtSIZ1 SUMO E3 ligase function [35] (Fig 1A), displayed morphological

The *siz1* mutant plants were smaller with shorter inflorescence stems compared to WT (Fig 1A). To determine whether *SIZ1* directly affects SCW formation, we employed an RNAi strategy to inhibit *AtSIZ1* expression specifically in the cells forming secondary walls. The promoter of the fiber cell-specific *SND1* was used to drive *SIZ1-RNAi* in *P*_*SND1*_*AtSIZ1-i* transgenic plants (S1A Fig). In transgenic lines, expression of *AtSIZ1* was suppressed by about 50% (S1E Fig). The wall thickness of the fiber cells in inflorescence stem was reduced compared to WT (S1B, S1C and S1F Fig). These suggest that *SIZ1* plays a role in SCW formation in inflorescence fiber cells.

**Fig 2:**
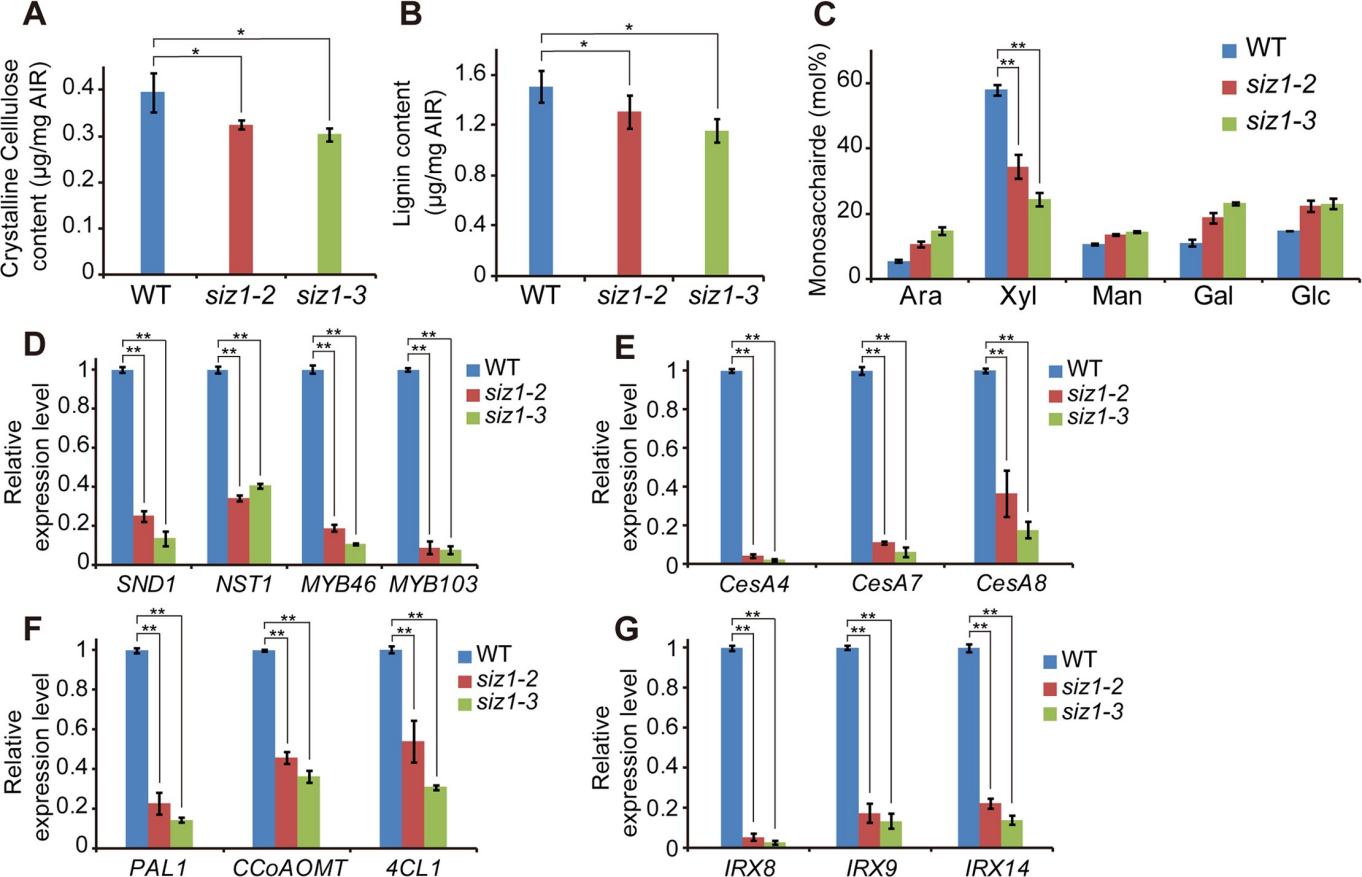
Reduction of SCW components and suppressed expression of genes related to SCW synthesis in *siz1* mutants. (A) Crystalline cellulose contents in wild type (WT) and *siz1* stems. (B) Lignin contents of WT and *siz1* stems. (C) Monosaccharide composition of cell wall residues from WT and *siz1* stems. (D)-(G) Expression level of SCW synthesis associated genes in the basal first and second internodes of inflorescence stem of WT and *siz1* plants. Relative levels of the indicated transcripts are normalized to *ACT2*. The WT transcript level of genes of interest was set to 1. Data represent average values±SD (n = 4 replicates in A-C, 3 in D-G). *P < 0.05, **P < 0.01 (Student`s *t*-test). AIR: Alcohol-insoluble residues.

To investigate how SCW formation is changed in the *siz1* mutants, we analyzed the chemical composition of their cell walls and examined expression of the SCW-related genes. In inflorescence stem crystalline cellulose and lignin were reduced by more than 20% in *siz1* plants compared to WT (Fig 2A and 2B).

The xylose from non-cellulosic polysaccharides was also significantly decreased in *siz1* plants (Fig 2C). Expression of the genes responsible for SCW formation was significantly suppressed in *siz1* plants. These genes included transcription factor genes (*SND1*, *NST1*, *MYB46* and *MYB103*)[1] (Fig 2D), SCW cellulose synthase genes (*CesA4*, *CesA7* and *CesA8*)[1] (Fig 2E), lignin biosynthesis genes (*PAL1*, *CCoAOMT* and *4CL1*)[1] (Fig 2F) and xylan biosynthesis genes (*IRX8*, *IRX9* and *IRX14*)[1] (Fig 2G). These results indicated that *AtSIZ1* is involved in regulating the transcriptional network that controls SCW formation.

### SIZ1 interacts with LBD30

**Fig 3:**
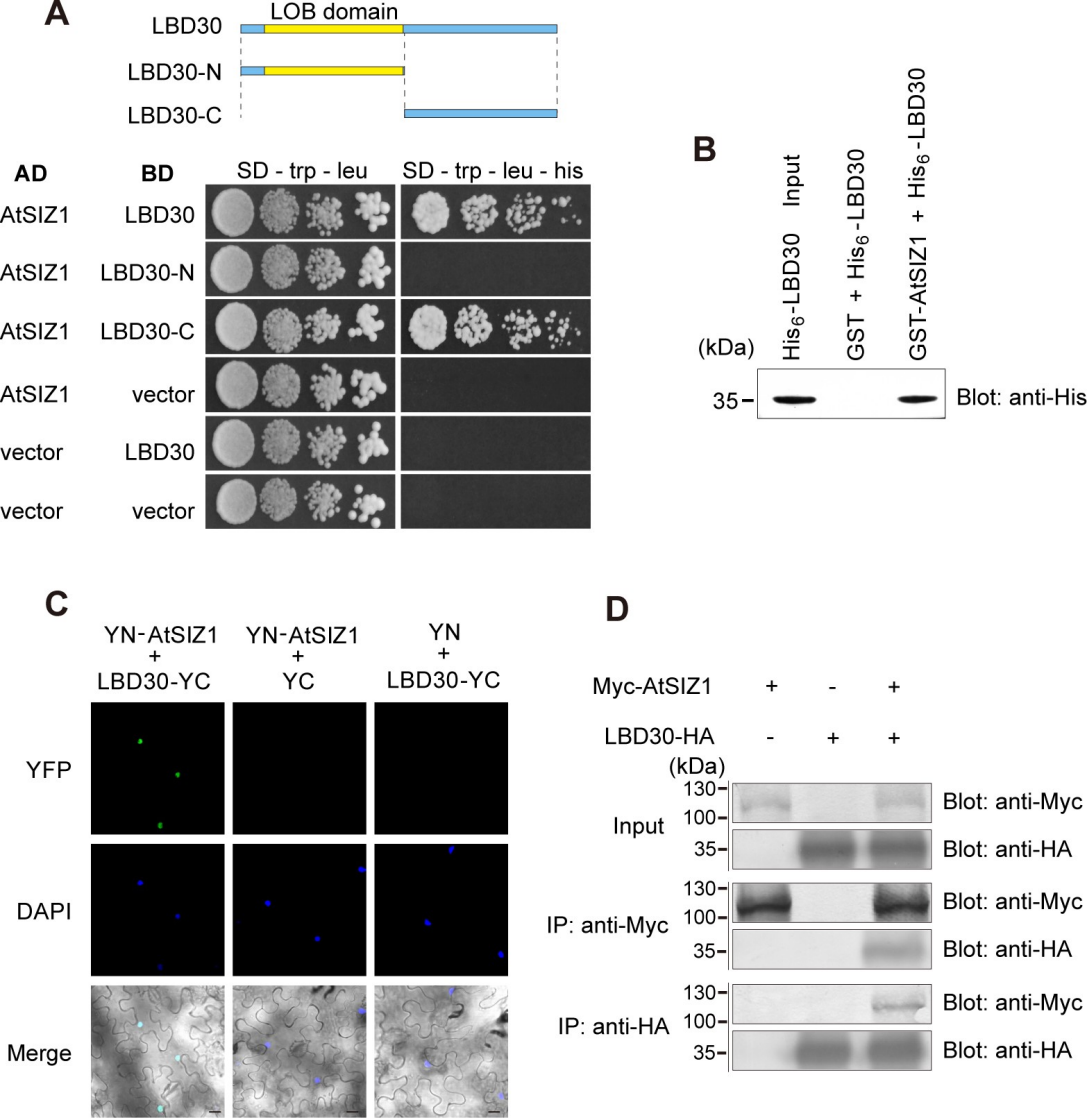
AtSIZ1 interacts with LBD30. (A) AtSIZ1 directly interacted with LBD30 through its C-terminus. Full-length *AtSIZ1* and various lengths of *LBD30* cDNA were fused to the Gal4 activation domain (AD) and the Gal4 DNA-binding domain (BD) and transformed into yeast cells in different combinations of empty vectors and recombinant plasmids. The upper panel indicates LBD30 and its derivates showing the conserved LOB domain (yellow). (B) *In vitro* pull-down of LBD30 with AtSIZ1. His_6_-LBD30 fusion protein pulled down with GST or GST-AtSIZ1 fusion protein was detected by immunoblotting using an anti-His antibody. (C) Bimolecular fluorescence complementation (BiFC) analysis of the interaction between AtSIZ1 and LBD30. Full-length *AtSIZ1* and *LBD30* cDNAs were fused to the N-terminal part of yellow fluorescent protein (YFP) and the C-terminal part of YFP, respectively. Infiltration of target protein with an empty vector was used as the negative control. DAPI (4′,6-diamidino-2-phenylindole) signal indicates nuclei. Scale bars = 20 μm. (D) Co-immunoprecipitation of AtSIZ1 and LBD30. Myc-tagged AtSIZ1 and HA-tagged LBD30 were expressed or coexpressed in tobacco leaves. Proteins were detected by immunoblotting with an anti-Myc antibody and an anti-HA antibody in crude lysates and in protein extracts after immunoprecipitation with an anti-Myc antibody and an anti-HA antibody, respectively. co-precipitation (Fig 3B) and bimolecular fluorescence complementation (BiFC) assays (Fig 3C). In addition, by co-expression of LBD30 and AtSIZ1 in tobacco (*N*. *benthamiana*) leaves, the two proteins interacted with each other (Fig 3D). Together, these results demonstrated that AtSIZ1 interacts with LBD30 at C-terminal.

*AtSIZ1* promoter was active in cortex cells and interfascicular fibers of the inflorescence stem undergoing SCW formation (S2 Fig). SIZ1 is a nuclear-localized protein [27] and functions in facilitating SUMO conjugation to target proteins [47]. Using AtSIZ1 as the bait against a cDNA library made from *A*. *thaliana* inflorescence stem undergoing SCW formation, we conducted yeast two-hybrid (Y2H) screening to identify its target proteins for sumoylation. Among 191 identified candidates, four were found to be different parts from the ASYMMETRIC LEAVES2/LATERAL ORGAN BOUNDARIES DOMAIN (AS2/LBD) protein, LBD30, encoded by At4g00220 locus [48, 49]. We examined *LBD30* expression in public databases and found that it is highly expressed in the inflorescence stem (S3 Fig). LBD/AS2 family proteins have a characteristic LOB domain at N terminus that possess DNA-binding ability [49, 50]. We re-examined the interaction between AtSIZ1 and LBD30 in an Y2H system and found that AtSIZ1 interacted with LBD30 through its C-terminus (LBD30-C, amino acids 121–228) (Fig 3A). This interaction was verified through

### AtSIZ1 mediates SUMO1 modification of LBD30

**Fig 4:**
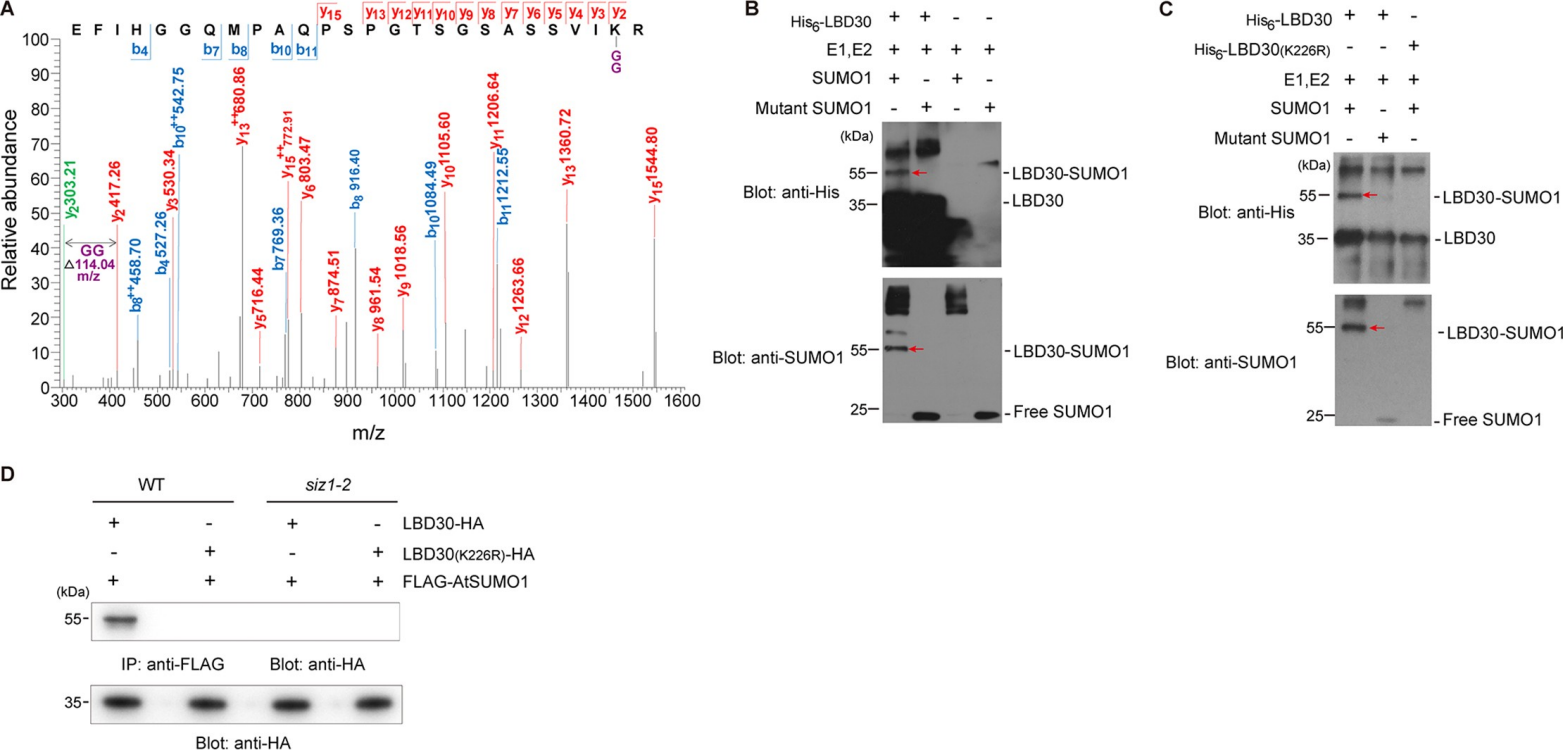
Identification of the LBD30 sumoylation site. (A) Tandem mass spectrometry (MS/MS) spectrum of the peptide fragment of LBD30 modified at Lysine226 by AtSUMO1. A modified AtSUMO1_(T91R)_ in which the residue preceding the C-terminal Gly-Gly (diGly) is replaced with a lysine was used, and digestion of the AtSUMO1_(T91R)_ protein conjugates with trypsin yielded a diGly motif attached to the target lysines. The observed y and b ions and the fragment map is shown. The fragment ions without modification are indicated in green. The diGly modification is reported in purple. (B, C) *In vitro* verification of LBD30 sumoylation. Recombinant His_6_-LBD30 or His_6_-LBD30(K226R) was used as a substrate in an *in vitro* sumoylation assay. Unconjugated and SUMO-conjugated LBD30 proteins were detected with anti-His and anti-SUMO1 antibody. (D) *In vivo* sumoylation of LBD30 in transgenic wild type and *siz1-2* plants overexpressing FLAG-AtSUMO1 and LBD30-HA or LBD30(K226R)-HA. Sumoylated LBD30 was detected by immunoblotting with an anti-HA antibody after immunoprecipitation with an anti-FLAG antibody. Red arrows indicate LBD30-SUMO1.

SIZ1 interacted with LBD30, but expression of *LBD30* was not altered in *siz1* mutants (S4 Fig). LBD30 is predicted to contain a sumoylation motif (ΨKXE) with K226 as a potential SUMO conjugation residue (S1 Table) and showed a high possibility to be sumoylated among a list of SCW formation-related proteins [1] (S1 Table). Then we examined whether LBD30 could be SUMO conjugated at the ΨKXE motif. Using tandem mass spectrometry analysis, a mutant AtSUMO1_(T91R)_ protein, which allows production of a signature peptide containing a diglycine remnant at the sumoylation site[51], was identified at K226 in LBD30 (Fig 4A). To verify this sumoylation, recombinant LBD30 was generated and the sumoylated LBD30 was detected in sumoylation assay (Fig 4B). When LBD30 was mutated to generate a K226R variant (LBD30_K226R_), the substitution of K226 to R resulted in failure of SUMO1 conjugation to LBD30 (Fig 4C) without affecting its nuclear localization (S5 Fig). Furthermore, we examined if the mutant LBD30 can be sumoylated by AtSIZ1 *in planta*. By combinational expression of LBD30 or LBD30_K226R_, AtSIZ1 and AtSUMO1 in tobacco leaves, immunoblotting indicated only LBD30 is SUMO-conjugated (S6 Fig). Next, AtSUMO1 and LBD30 or LBD30_K226R_ were co-expressed in *siz1-2* and WT *A*. *thaliana*. AtSUMO1 conjugation to LBD30 was only detected in the transgenic plants with WT background expressing LBD30 and AtSUMO1 (Fig 4D). These demonstrated that SIZ1 mediates LBD30 sumoylation at the K226 residue.

### AtSIZ1-mediated sumoylation of LBD30 affects SCW formation and development

**Fig 5:**
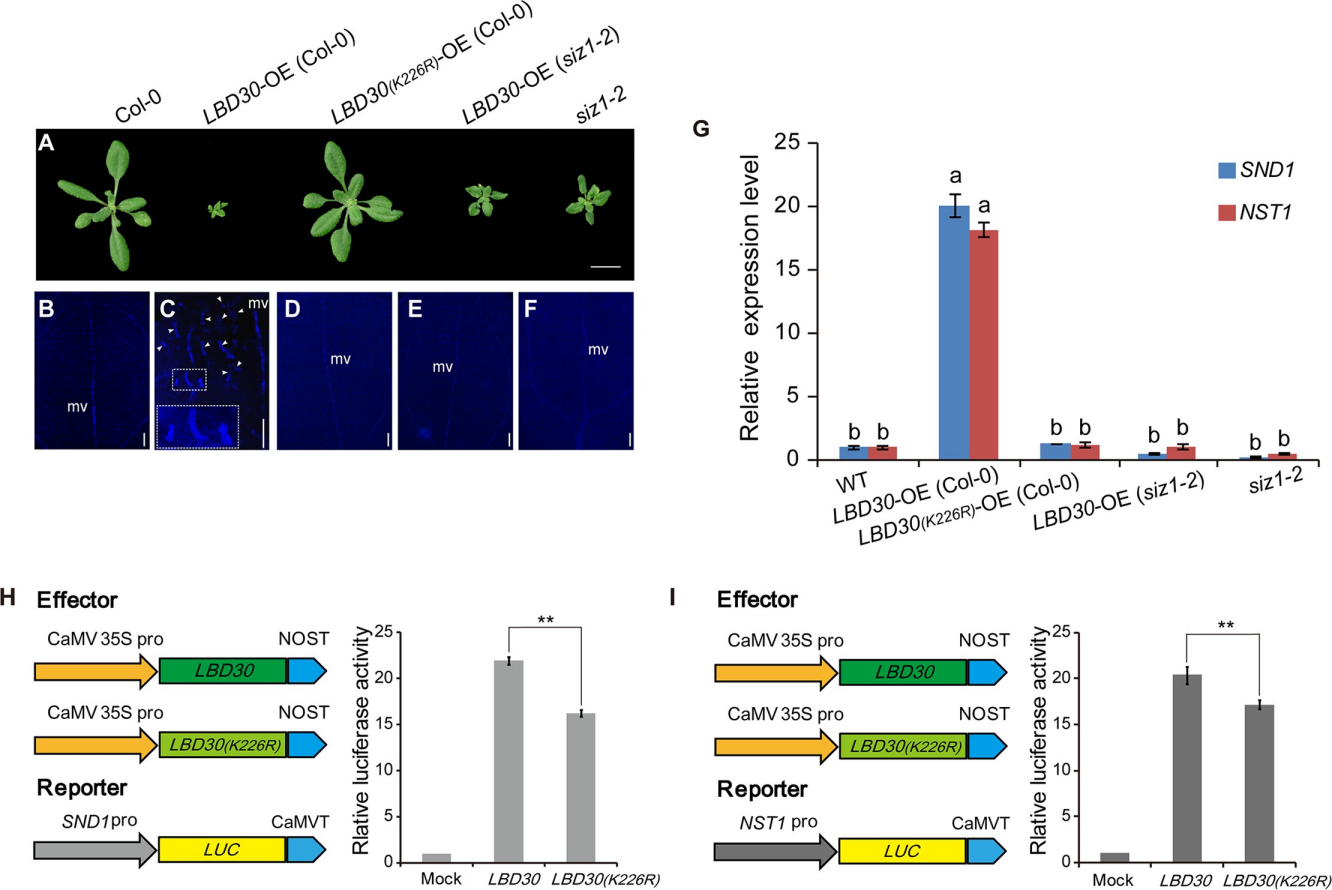
Overexpression of sumoylated LBD30 affects plant development and induce ectopic deposition of lignified SCWs. (A) 2-weeks old seedlings of the wild type (WT, Col-0), an LBD30 overexpressing transgenic plant, an LBD30_(K226R)_ overexpressing transgenic plant, an LBD30 overexpressing transgenic plant in the *siz1-2* mutant background and the *siz1-2* mutant (Col-0). (B-F) Lignin autofluorescent signals of cotyledons of plants corresponding to (A). White arrowheads show ectopic secondary wall thickening in the cotyledon of the LBD30 overexpressing transgenic plant. White dotted box shows a higher magnification picture. mv, middle vein. (G) Expression level of *SND1* and *NST1* in whole seedlings of WT, mutants and transgenic *Arabidopsis thaliana* plants. Relative levels are normalized to *ACT2*. The WT transcript level of genes of interest were set to 1. Data represent average values±SD (n = 3 replicates). Lowercase letters indicate significant differences at p < 0.01 by ANOVA. (H-I) Transcriptional activation analysis of *SND1* (H) and *NST1* (I) by LBD30 or LBD30_(K226R)_ in *A*. *thaliana* protoplasts. Data represent average values±SD (n = 3 replicates). **P < 0.01(Student`s *t*-test). Bars = 10 mm in (A), 200 μm in (B to F).

We investigated the effect of LBD30 sumoylation in the transgenics overexpressing *LBD30* and *LBD30*_*(K226R)*_. Overexpression of *LBD30* caused drastic phenotypic changes, severe dwarfism, short petioles and downward curled leaves. Ectopic lignin deposition was detected in cotyledons in 24 out of 28 T1 transgenic plants (Fig 5A–5C). In contrast, overexpression of *LBD30*_*K226R*_ showed little phenotypic changes (Fig 5A and 5D). Similarly, expression of *LBD30* in *siz1*-2 caused no phenotypic change in 28 transgenic plants out of 36 T1 plants and minor changes in remaining 8 plants compared to the *siz1-2* plants (Fig 5A, 5E and 5F). LBD30 sumoylation played a role in development and secondary cell wall biosynthesis.

Then, we investigated whether the SCW defects in *siz1* plants is caused by failure of LBD30 SUMO modification. We examined the transcripts of *SND1* and *NST1* in the transgenics overexpressing *LBD30* (S7A Fig). The transgenic plants were unable to develop normal inflorescence stem (Fig 5A) but expression of *SND1* and *NST1* was drastically up-regulated in the 2 weeks-old seedlings (Fig 5G). This upregulation of *SND1* and *NST1* expression was insignificant in the transgenics carrying *LBD30*_*K226R*_ or in the transgenics overexpressing *LBD30* in *siz1* mutant background (Fig 5G). When *LBD30* was overexpressed in *nst1/snd1* double mutant[11–13], ectopic lignin deposition in cotyledons was not detected in the transgenics (31/31 T1 plants) and the *nst1/snd1* double mutant (S7B and S7C Fig). On the other hand, we evaluated the effect of LBD30 sumoylation on *SND1* and *NST1* expression using a dual luciferase assay in *A*. *thaliana* protoplasts. The effecter was constructed by using a 35S promoter to drive expression of *LBD30* and *LBD30*_*(K226R)*_. A firefly luciferase driven by *SND1* or *NST1* promoter was used as a reporter (Fig 5H and 5I). LBD30 showed a significantly higher activity in activation of *SND1* or *NST1* promoter than LBD30_(K226R)_ (Fig 5H and 5I), suggesting that LBD30 SUMO conjugation affected *SND1* and *NST1* expression. Thus, the SUMO modification of LBD30 played a role in regulating SCW formation through the *SND1*/*NST1*-directed transcriptional network.

## Discussion

In higher plants all cells form primary cell wall. In some type cells, additional SCWs are formed inside the primary wall, providing plants with mechanical support for erect growth and channels for long-distance transportation of water, nutrients, and photosynthetic products. Formation of the SCWs in various type cells need to be precisely regulated in a spatio-temporal manner during growth and development [52]. To ensure a precise deposition of SCWs in some type cells, multiple levels of regulation have to be developed in plants. Disturbance of the regulatory networks causes abnormal growth and development [1]. At the transcriptional level, complex regulatory networks are involved in SCW formation [8, 53]. SCW formation in different cell types is initiated through cell type-specific transcription regulators [11–15]. Many signaling molecules regulating SCW formation have yet-to-be characterized [54].

At the protein level, post-translation modifications, such as protein phosphorylation and N-glycosylation [16, 17], are also being studied for their roles in regulating SCW formation. While a large number of proteins are modified with SUMO-conjugation and such modification affects a variety of biological processes [18], this study presents a detailed picture of how sumolyation can lead to the upregulation of SCW formation. Specifically, we found LBD30 sumoylation is required for activation of the *SND1/NST1*-mediated transcriptional networks in SCW formation.

*AtSIZ1*-mediated sumoylation is involved in a variety of growth and development processes such as flowering, response to light, immunity and nutrient element metabolisms in *A*. *thaliana* [31–34, 36, 55]. In this study, we observed that *siz1* mutants displayed defective SCWs in interfascicular fiber cells. Analysis indicated that SIZ1 interacted with LBD30 and catalyzed its sumoylation at K226 position in the sumoylation motif.

LBD30 is a transcription factor belonging to the Lateral Organ Boundaries Domain (LBD) family[56, 57]. *LBD30* and its homolog *LBD18* in *A*. *thaliana* were preferentially expressed in vascular tissues and *LBD18* played a role in regulating tracheary element differentiation[57].

**Fig 6:**
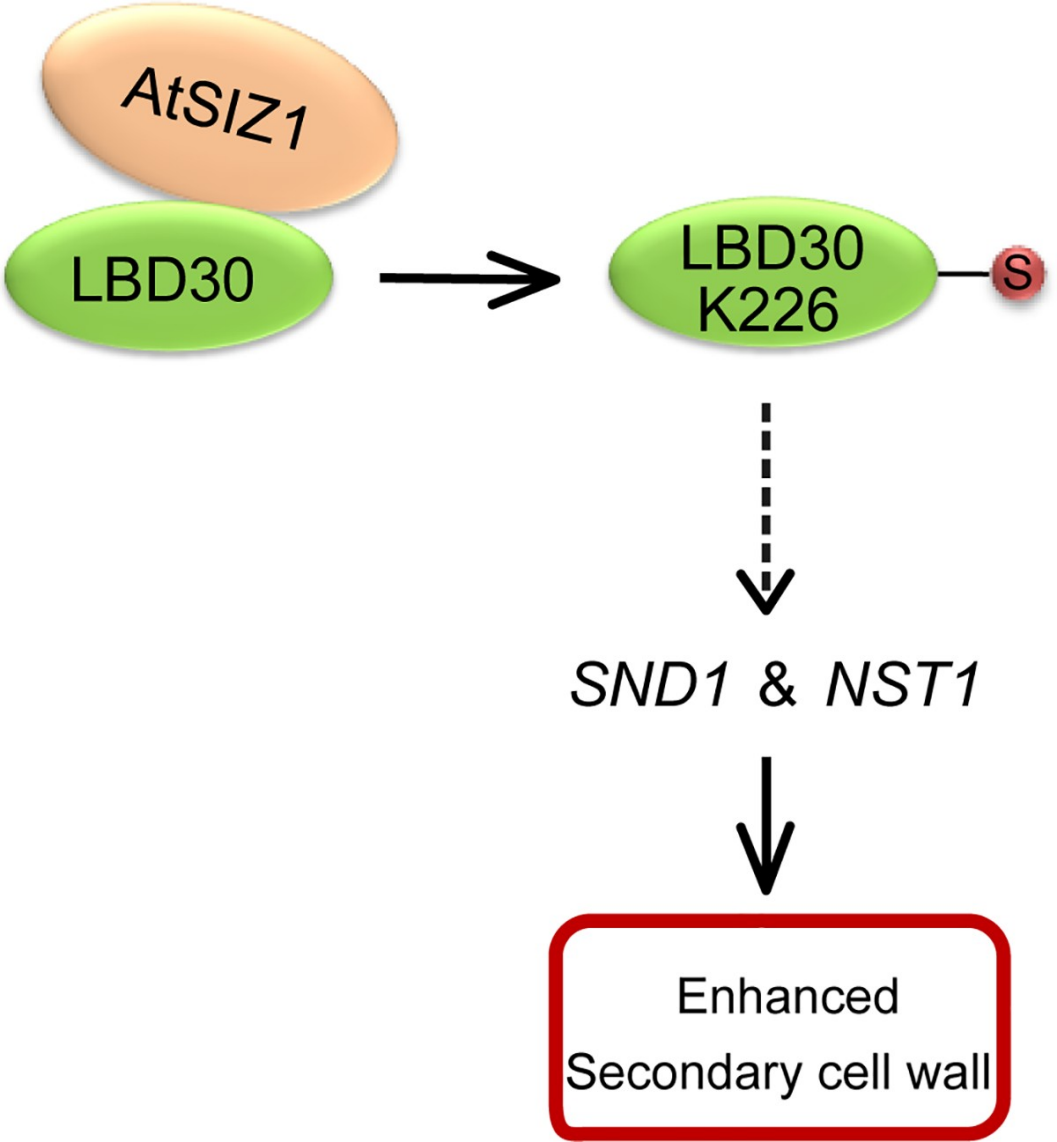
A model of the role of sumoylation in the regulation of SCW formation. AtSIZ1 catalyzes the sumoylation of LBD30 at K226. SUMO conjugation to LBD30 facilitates the expression of SND1 and NST1, and the SCW accumulates in cell types undergoing SCW thickening.

Defective SIZ1 or mutated LBD30 at K226 position led to loss of LBD30 function during the formation of SCW in interfascicular fiber cells. The evidence indicated that LBD30, when it was sumoylated by SIZ1, played a role in activating the *SND1*/*NST1*-mediated transcriptional networks (Fig 6) which regulate SCW formation in the fiber cells of inflorescence stem.

Generally, stress conditions cause activation of SCW formation [58, 59]. Several transcription factors sumoylated by AtSIZ1 are related to stress responses, including ICE1 in freezing stress[39], HsfA2 in heat stress[60], PHR1 in phosphate (Pi) deficiency[35], MYB30 and ABI5 in the abscisic acid-dependent drought stress[42, 43]. It is worthy of further study whether LBD30 sumoylation acts as a linking device between stress responses and SCW formation.

Generally LBD family proteins regulate plant development through interaction with other transcription factors [50]. A number of transcription factors have been identified to bind to *SND1* and *NST1* promoters to activate their expression[1, 61]. In this study, we found that transcription factor LBD30 was sumoylated by SIZ1 and such protein modification affected activation of the *SND1*/*NST1*-mediated transcriptional networks for SCW formation in fiber cells. Though it remains to be investigated how LBD30 sumoylation performs its function in activation of the transcriptional networks, one possibility is that LBD30 sumoylation may affect the transcription factor interactions that are necessary for activation of *SND1/NST1* expression. This possibility might justify the observation that LBD30 sumoylation showed different strength of effect on *SND1* and *NST1* expression between transgenics and protoplast system. Interaction of LBD30 with other factors in *planta* affected the *SND1* and *NST1* promoter activity.

The finding that LBD30 sumoylation acts as another layer of regulation to aid in the precise control of SCW formation provides additional insight into a key process that is essential for upright growth and the long-distance transport of water and solutes in plants and has implications in cell wall modification via regulation of LBD30 sumoylation in crop improvement.

## Materials and methods

### Plant materials and culture conditions

The *A*. *thaliana* Col-0 ecotype (WT) and the T-DNA insertion mutant lines, *siz1-2* (SALK_065397) [35], *siz1-3* (SALK_034008) [35] and *snd1/nst1* double mutant (CS67921) [11–13], were grown in a phytotron at 22°C with a photoperiod of 16 h of light and 8 h of darkness. Transformation of *A*. *thaliana* was performed using the *Agrobacterium tumefaciens*-mediated floral dip method [62]. Transgenic plants were selected on MS medium containing 50 μg/ml hygromycin. Positive T2 transgenic plants were used for further analysis, with the exception of *LBD30* overexpressing plants in the Col-0 background, where T1 plants were used because the T1 transgenic displayed severe growth defects and hardly produced seeds.

### Gene cloning and plasmid construction

cDNAs for *AtSIZ1* (At5g60410), *LBD30* (At4g00220), *AtSUMO1* (At4g26840) and the promoter regions of AtSIZ1(3535bp), SND1(2858bp) and NST1(2913bp) were PCR-amplified from a cDNA pool of *A*. *thaliana* as well as from genomic DNA with specific primers listed in S2 Table. For the Y2H assay, the coding region of LBD30 and AtSIZ1 were inserted respectively into the pGBKT7 and pGADT7 plasmids (Clontech) and introduced into AH109 yeast cells (Clontech) following the manual. For BiFC analysis, LBD30-YC and YN-AtSIZ1 were constructed as previously described [63] and mobilized into *A*. *tumefaciens* strain GV3101 and transformed into *Nicotiana benthamiana* tobacco leaf cells [63]. For purification of recombinant proteins, the *LBD30*, a mutated LBD30_(K226R)_ and *AtSIZ1* coding regions were cloned into the pET-28b (Novagen) and pGEX-4T-1 (GE Healthcare) plasmids to produce the His_6_-LBD30, His_6_-LBD30_(K226R)_ and GST-AtSIZ1 fusion proteins, respectively. The site directed mutagenesis of LBD30(K226R) was generated according to Hieff Mut^TM^ Site-Directed Mutagenesis Kit (Yeasen Biotech). For protein expression in plants, the full coding regions of *AtSIZ1*, *LBD30*, *LBD30*_*(K226R)*_ and *AtSUMO1* were subcloned into the binary pCambia 1300 vector to produce chimeric MYC-AtSIZ1, LBD30-HA, LBD30_(K226R)_-HA and FLAG-AtSUMO1 fusions under the control of the constitutive CaMV 35S promoter. These constructs were coexpressed in *Nicotiana benthamiana* tobacco leaves for transient expression, and transformed into *A*. *thaliana* to produce stably transformed plants. Constructs encoding LBD30 or LBD30_(K226R)_ and green fluorescent protein (GFP) fusion proteins under the control of the CaMV 35S promoter were generated and introduced into *A*. *thaliana* protoplasts [64] to investigate subcellular localization.

For transcriptional activation analysis, the coding regions of *LBD30* and *LBD30*_*(K226R)*_ and the promoter regions of the *SND1* and *NST1* genes were cloned into the effector (35S-transcription factor) and reporter (firefly luciferase) vectors (pGreenII vector, Promega) and then coexpressed in *A*. *thaliana* protoplasts[64]. For analysis of *AtSIZ1* expression, an *AtSIZ1* promoter fragment was cloned and fused to a β-glucuronidase (GUS) reporter gene in the pCambia1301 vector for *A*. *thaliana* transformation. To investigate the function of *AtSIZ1* in inflorescence stems, two different genomic DNA fragments specific to AtSIZ1 were amplified separately to form hairpin structures under the control of the *SND1* gene promoter. These constructs were designed to cause RNAi suppression (*SND1*promoter-*AtSIZ1*RNAi1 and *SND1*promoter-*AtSIZ1*RNAi2) specifically in *A*. *thaliana* inflorescence stems.

### Microscopy analyses

The basal internodes of inflorescence stems of 8-week-old plants with the same flowering date were collected as described before. Briefly, the internodes were fixed in FAA overnight and embedded in paraffin (Sigma-Aldrich 18635) after dehydration through a graded ethanol series. Ten-micrometer-thick sections were cut and stained with toluidine blue for light microscopy. Free-hand cross sections of *A*. *thaliana* inflorescence stems were stained with 0.5% phloroglucinol (Sigma-Aldrich P3502) (w/v) in 12% HCl for 3 min, and immediately observed under a bright-field microscope (OLYMPUS BX53). For transmission electron microscopy, ultrathin sections were cut and observed as described [65]. To visualize lignin auto-fluorescence under UV light and the sub-cellular localization of GFP-fusion proteins, *A*. *thaliana* cotyledons were grown on MS plates and *A*. *thaliana* leaf protoplasts were observed using a fluorescent microscope (OLYMPUS BX53). For the BiFC analysis, tobacco leaf cells were stained with DAPI [66]and visualized using a confocal microscope (LSM 510 META; Zeiss).

### Analysis of cell wall components

Fluorescence stems from at least three independent 8-week-old *A*. *thaliana* WT or mutant plants were collected and ground in liquid nitrogen to a fine powder to prepare alcohol insoluble residue (AIR) as previously described [67]. After the de-starched procedure [67], the crystalline cellulose content and monosaccharide composition were analyzed according to a previously published protocol [68]. The lignin content was determined following the methods in [69].

### Gene expression analysis

Total RNA isolated from the lower center part of the inflorescence stem of 4-week-old *A*. *thaliana* plants and whole seedlings of 2-week-old WT, mutants and transgenic plants were extracted using the E.Z.N.A. Total RNA Kit (Omega) according to the manufacturer’s instructions. cDNA was synthesized by treatment with reverse transcriptase and oligo (dT) primer (TransScript One-Step gDNA Removal and cDNA Synthesis SuperMix, Transgene Biotech) and quantitative PCR assays were conducted with a MyiQ real-time PCR detection system (Bio-Rad) using SYBR Green (TransStart Top Green qPCR MIX) following the user manual. The *A*. *thaliana ACT2* gene (AT3G18780) was used as an internal control to normalize the data. The mathematical analysis for qPCR quantification was delta-delta Ct method [70]. The quantitative PCR (qPCR) experiment was performed in biological triplicates.

### GUS staining assay

Free-hand cross-sections of the lower internodes of the inflorescence stems from 4 week old *AtSIZ* promoter-GUS transgenic *A*. *thaliana* were examined for GUS activity as previously described [71].

### Protein-protein interaction assay

To identify AtSIZ1 interacting proteins, a Y2H library was generated using cDNA derived from 4-week-old *A*. *thaliana* inflorescence stems and used to screen for target proteins, using the Make Your Own Mate & Plate Library System (Clontech), according to the manufacturer`s directions. For the BiFC analysis, the constructs were transformed into *Agrobacterium* strain GV3101, and the resulting strains were used to transform *N*. *benthamiana* leaf cells, either individually or in combination. The leaves were examined after 48 h of incubation. To investigate the physical interaction between AtSIZ1 and LBD30 *in vitro*, recombinant His_6_-LBD30 and GST-AtSIZ1 proteins were expressed in *Escherichia coli* and purified with Ni-NTA Agarose (Qiagen) and Pierce GST Agarose (Thermo Scientific), according to the manufacturer’s instructions. GST and GST-tagged AtSIZ1 proteins from the cell lysates were first immobilized on the GST Agarose (Thermo Scientific). After washing away unbound proteins with 1×PBS, the immobilized GST and GST-AtSIZ1 proteins were incubated with the cell lysate of *Escherichia coli* expressing His_6_-LBD30. After several washing steps with 1×PBS, the complexes were eluted with 2×SDS loading buffer and boiled at 100°C for 5 min. The eluted proteins were separated by SDS-PAGE, transferred to a PVDF membrane and the protein was immunoblotted with an anti-His antibody (1:5000 dilution, Abmart).

*In vivo* AtSIZ1 and LBD30 interactions were analyzed by co-immunoprecipitation (co-IP). Myc-tagged AtSIZ1 and HA-tagged LBD30 were expressed transiently in tobacco leaf cells. Proteins were extracted by grinding the leaves in liquid nitrogen and thawed in extraction buffer [50 mM Tris-HCl, pH 7.5, 150 mM NaCl, 1mM EDTA, 0.2% Triton X-100 (Sigma Aldrich), 10% glycerol, 1mM PMSF, 2% PVPP (polyvinylpolypyrrolidone) and 1x concentration of protease inhibitor cocktail (Roche)] for 30 min. The homogenate was then filtered through a 0.22 μm filter membrane (Millipore) and 1mL of the filtrate was incubated with 50 μL agarose conjugated anti-Myc mouse monoclonal antibody (Abmart) or 50 μL anti-HA rat monoclonal antibody Affinity Matrix (Roche) for 3 h at 4°C. The beads were washed three times with wash buffer (50 mM Tris-HCl, pH 7.5, 150 mM NaCl, 1 mM EDTA, 0.2% Triton X-100), and the bound proteins were eluted with 2×SDS-PAGE loading buffer and boiled at 100°C for 5 min. The eluted proteins were immunoblotted as above and incubated with anti-HA mouse monoclonal antibody (1:3000 dilution, Abmart) or anti-Myc mouse monoclonal antibody (1:3000 dilution, Abmart).

### Sumoylation assay

The *in vitro* sumoylation was performed using the SUMOlink^TM^ SUMO-1 Kit (Active Motif). Briefly, recombinant His_6_-LBD30 and His_6_-LBD30_(K226R)_ proteins were expressed in *E*. *coli* and purified. A total of 3 μg of target protein was added to 20 μl reaction buffer and incubated at 30°C for 3 h. The reaction was stopped by adding 10 μl of 2×SDS-PAGE loading buffer. Sumoylated of His_6_-LBD30 was detected by immunoblot analysis using an anti-His mouse monoclonal antibody (1:5000 dilution, Abmart) and a SUMO-1 rabbit antibody (1:2000 dilution, Active Motif).

The reaction mixture was also separated by SDS-PAGE. After staining with Coomassie Blue R-250, the sumoylated His_6_-LBD30 protein band was cut into 1 mm wide pieces for digestion and liquid chromatography-tandem mass spectrometry (LC-MS ⁄MS) analysis of the LBD30 sumoylation site. Protein digestion for LC-MS/MS analysis was performed by the Beijing Protein Institute. Briefly, the protein bands were destained with 50% v/v acetonitrile (ACN) [72] and 25 mM ammonium bicarbonate and dried in 100% ACN and the gel slices were incubated with a 10 ng μl^-1^ trypsin solution in 25 mM ammonium bicarbonate at 37°C for 12h. The extracts were then dried in a stream of N_2_ and resuspended in 5% ACN in 0.1% v/v formic acid FA. LC-MS ⁄MS analysis was performed using an Ultimate3000 liquid chromatography system (Dionex) connected to a Q Exactive mass spectrometer (Thermo Scientific) as decribed previously [72] with modifications. The extracts were separated by a C18 reverse-phase column with a 1 hour gradient of mobile phase (phase A, 5% ACN in 0.1% FA; phase B, 95% CAN in 0.1% FA) at a flow rate of 300 nL / min. The separated sample was then injected into the mass spectrometer and a method of full scans were acquired with AGC target value of 1E6, resolution of 70,000 FWHM at 200 m/z, and maximum ion injection time (IT) of 100 ms. The mass spectura were extracted by BioWork version 3.3.1 sp1 (Thermo Fisher). All MS/MS samples were analyzed using Mascot software (Marix Science).

For the *in vivo* sumoylation assay, the Myc tagged AtSIZ1, the FLAG-tagged AtSUMO1 and the HA-tagged LBD30 or LBD30_(K226R)_ were expressed in tobacco leaves. The FLAG-AtSUMO1 transgenic *A*. *thaliana* plants (Col-0 background) were crossed with LBD30-HA or LBD30_(K226R)_-HA transgenic *A*. *thaliana* plants (*siz1-2* background). F2 progeny of transgenic plants with WT and *siz1-2* background overexpressing FLAG-AtSUMO1 and LBD30-HA or LBD30_(K226R)-_HA were obtained. Total proteins were extracted and immunoprecipitated with an anti-FLAG mouse monoclonal M2 affinity gel (Sigma-Aldrich). The sumoylated LBD30 was detected by immunoblotting with an anti-HA Rat monoclonal high-affinity antibody (1:2000 dilution, Roche) after IP.

### Dual luciferase assay

Protoplasts used in the transient effector-reporter analysis were isolated from 2-week-old *A*. *thaliana* seedlings as previously described [64]. The coding sequences of LBD30 and LBD30_(K226R)_ were cloned into the effector plasmid. The promoters of *SND1* and *NST1* were cloned into the firefly luciferase reporter vector (pGreenII, Promega). The Renilla luciferase gene driven by the CaMV 35S promoter served as a control to normalize for transformation efficiency. Luciferase activities were measured with a dual-luciferase reporter assay system (Promega).

## Supporting information

S1 Fig Reduction in SCW thickness by RNAi repression of *SIZ1* in cell types that undergo SCW thickening. (A) Wild type (WT, Col-0) plant (left), transgenic *Arabidopsis thaliana* plants with *SND1* promoter controlled RNAi inhibition of *SIZ1* (middle) and *siz1* mutants (right). (B-D) Cross sections of WT and *SIZ1* RNAi transgenic plant stems stained with phloroglucinol-HCl. if: interfascicular fiber, ve: vessel, xf: xylary fiber. Scale bars = 10 mm in (A), 20 μm in (B-D). (E) Quantitative PCR analysis showing a reduction in the mRNA levels of *SIZ1* in the stems of two independent *SND1* promoter-*SIZ1* RNAi lines. The expression level of *SIZ1* in Col-0 was set to 1. Data represent average values±SD (n = 3). **P < 0.01(Student`s *t*-test). (F) Wall thickness of vessels and fibers in the inflorescence stems of WT and transgenic plants. Data represent average values±SD (n = 30 cells from 3 independent plants). **P < 0.01(Student`s *t*-test).
(TIF)

S2 Fig *SIZ1* promoter activity in inflorescence stems. (A) *SIZ1* promoter-GUS (β-glucuronidase) expression in a cross-section of an internode near the cessation of elongation in an inflorescence stem of a 4 weeks old transgenic *Arabidopsis thaliana* plant. (B) High magnification of a stem section of a *SIZ1* promoter-GUS transgenic plant. co: cortex, if: interfascicular fiber, vb: vascular bundle. Scale bars = 100 μm in (A), 20 μm in (B).
(TIF)

S3 Fig LBD30 expression profile during *Arabidopsis thaliana* development. The region indicated by the black line is the second internode of the inflorescence stem. Data were obtained from the AtGenExpress Visualization Tool (http://jsp.weigelworld.org/expviz/expviz.jsp).
(TIF)

S4 Fig The *LBD30* expression level in the basal first and second internodes of inflorescence stems of wild-type (WT) and *siz1* plants. Relative levels were normalized to *ACT2*. The transcript level of *LBD30* in WT was set to 1.0. Data represent average values±SD (n = 3 replicates).
(TIF)

S5 Fig Fluorescent signals of green fluorescent protein (GFP) fused LBD30 proteins in *Arabidopsis thaliana* leaf protoplasts. (A) A protoplast expressing GFP alone. (B, C) Protoplasts expressing GFP tagged LBD30. (D, E) Protoplasts expressing GFP tagged LBD30(K226R).
(TIF)

S6 Fig Examination of LBD30 sumoylation in tobacco. Myc tagged AtSIZ1, FLAG-tagged AtSUMO1, and HA-tagged LBD30 or LBD30_(K226R)_ were expressed in tobacco leaves as indicated. Expression of the proteins was detected by anti-Myc, anti-HA and anti-FLAG antibodies, respectively. After immunoprecipitation with an anti-FLAG antibody, sumoylated LBD30 was detected by immunoblotting with an anti-HA antibody. Black arrows indicate Myc-AtSIZ1.
(TIF)

S7 Fig Immunoblot detection of LBD30-cHA expression. **(A)** The protein expression level of LBD30-cHA. ACTIN was used as an internal control, detected by an anti-ACTIN antibody (1:3000 dilution, Abmart). (B,C) Lignin autofluorescent signals of cotyledons of LBD30 overexpressing transgenic plant in the *nst1/snd1* double mutant background and the *nst1/snd1* double mutant (Col-0). mv, middle vein. Bars = 200 μm in (B to F).
(TIF)

S1 Table Predicted sumoylation sites in SCW related proteins by GPS-SUMO. (XLS)

S2 Table Primers used in this study. (DOCX)
