# Supplementary figures and images for: SUMO modification of LBD30 by SIZ1 regulates secondary cell wall formation in Arabidopsis thaliana
Source: PLoS Genet. 2019 Jan 18;15(1):e1007928. doi: 10.1371/journal.pgen.1007928 (PMC6355022; doi:10.1371/journal.pgen.1007928)

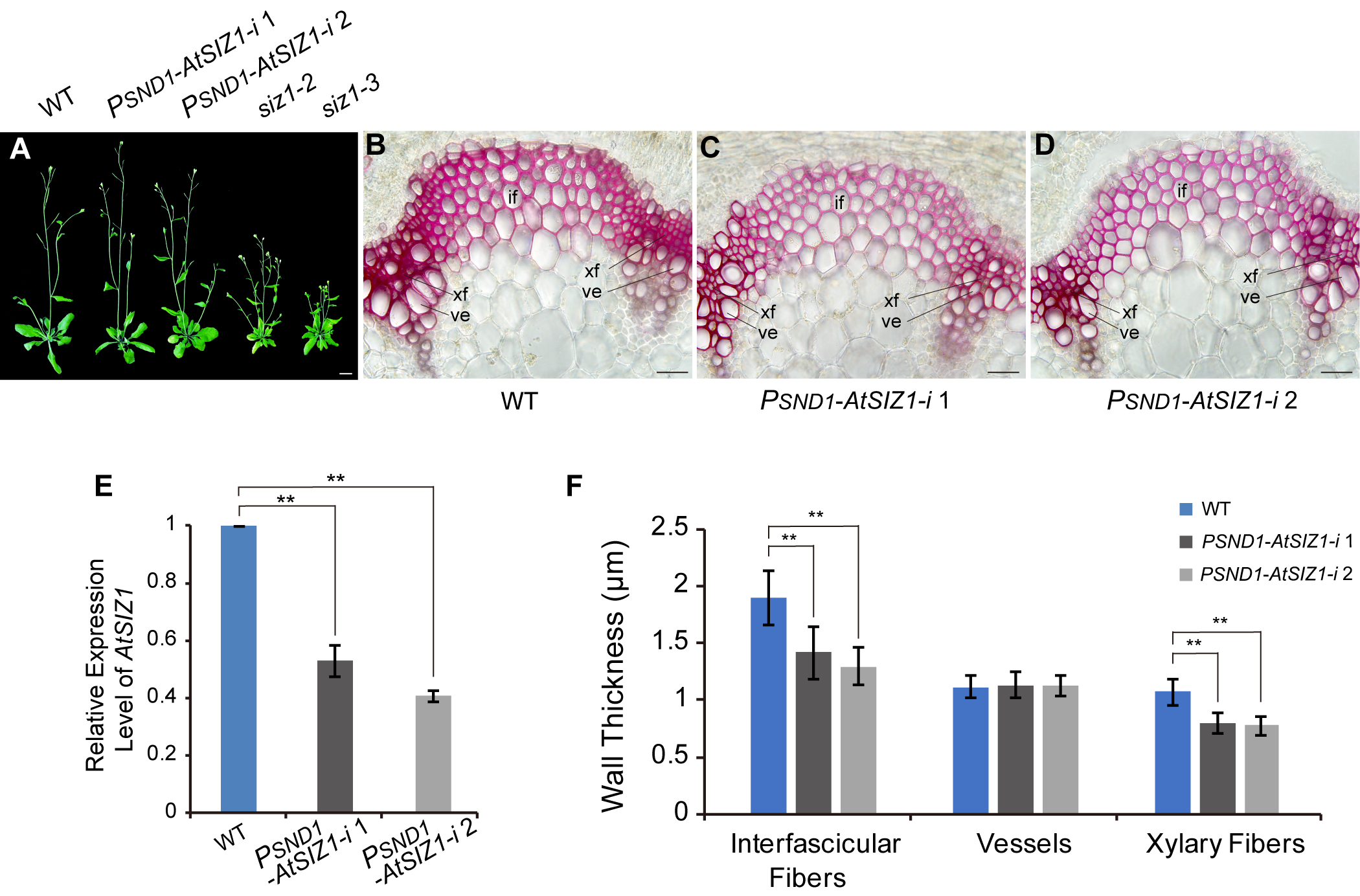

Supplement: S1 Fig — (A) Wild type (WT, Col-0) plant (left), transgenic Arabidopsis thaliana plants with SND1 promoter controlled RNAi inhibition of SIZ1 (middle) and siz1 mutants (right). (B-D) Cross sections of WT and SIZ1 RNAi transgenic plant stems stained with phloroglucinol-HCl. if: interfascicular fiber, ve: vessel, xf: xylary fiber. Scale bars = 10 mm in (A), 20 μm in (B-D). (E) Quantitative PCR analysis showing a reduction in the mRNA levels of SIZ1 in the stems of two independent SND1 promoter-SIZ1 RNAi lines. The expression level of SIZ1 in Col-0 was set to 1. Data represent average values±SD (n = 3). **P < 0.01(Student`s t-test). (F) Wall thickness of vessels and fibers in the inflorescence stems of WT and transgenic plants. Data represent average values±SD (n = 30 cells from 3 independent plants). **P < 0.01(Student`s t-test). (TIF) [file pgen.1007928.s001.tif]

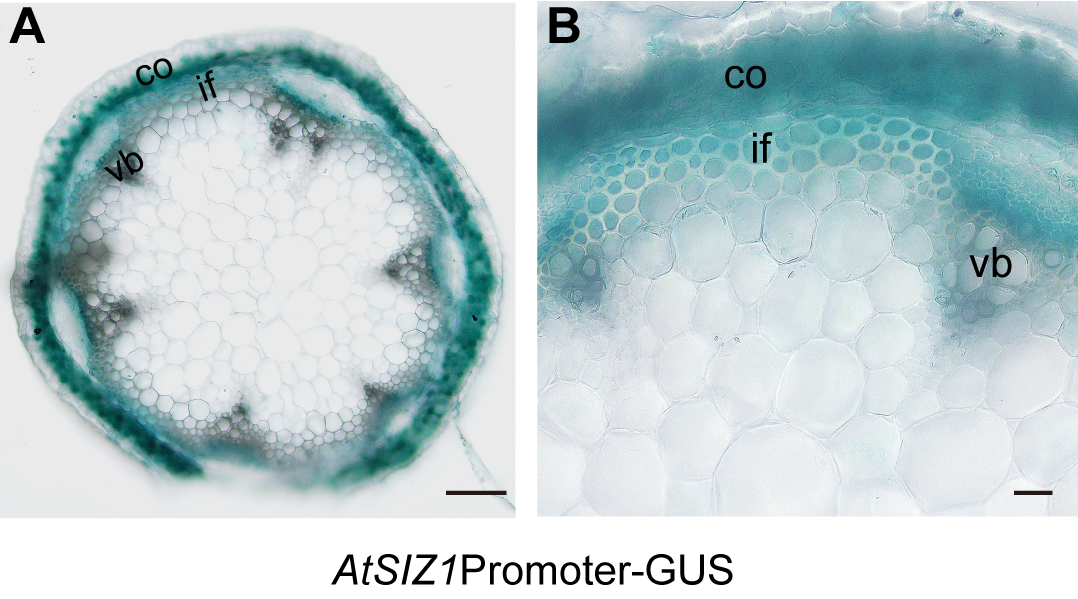

Supplement: S2 Fig — (A) SIZ1 promoter-GUS (β-glucuronidase) expression in a cross-section of an internode near the cessation of elongation in an inflorescence stem of a 4 weeks old transgenic Arabidopsis thaliana plant. (B) High magnification of a stem section of a SIZ1 promoter-GUS transgenic plant. co: cortex, if: interfascicular fiber, vb: vascular bundle. Scale bars = 100 μm in (A), 20 μm in (B). (TIF) [file pgen.1007928.s002.tif]

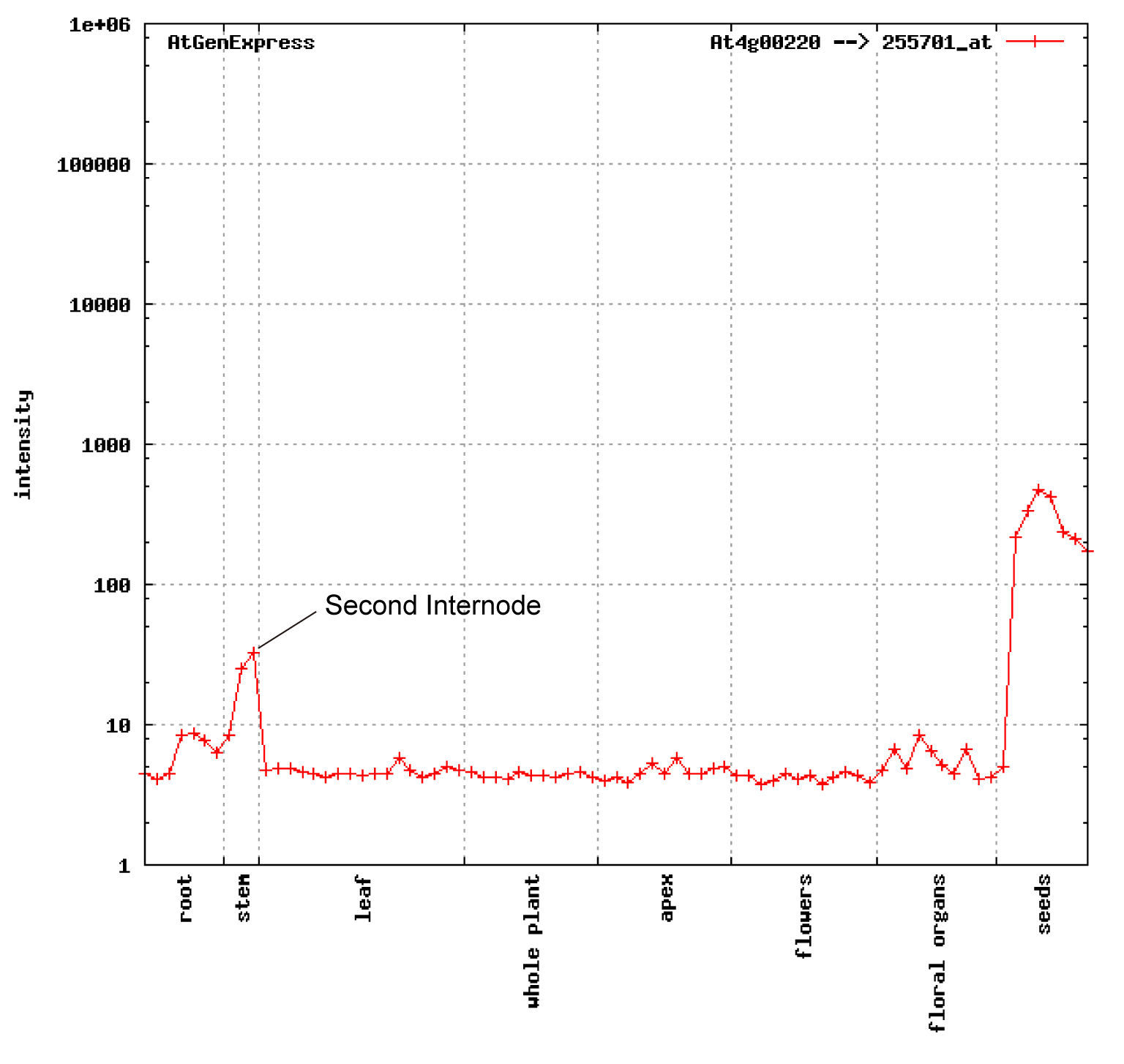

Supplement: S3 Fig — The region indicated by the black line is the second internode of the inflorescence stem. Data were obtained from the AtGenExpress Visualization Tool (http://jsp.weigelworld.org/expviz/expviz.jsp). (TIF) [file pgen.1007928.s003.tif]

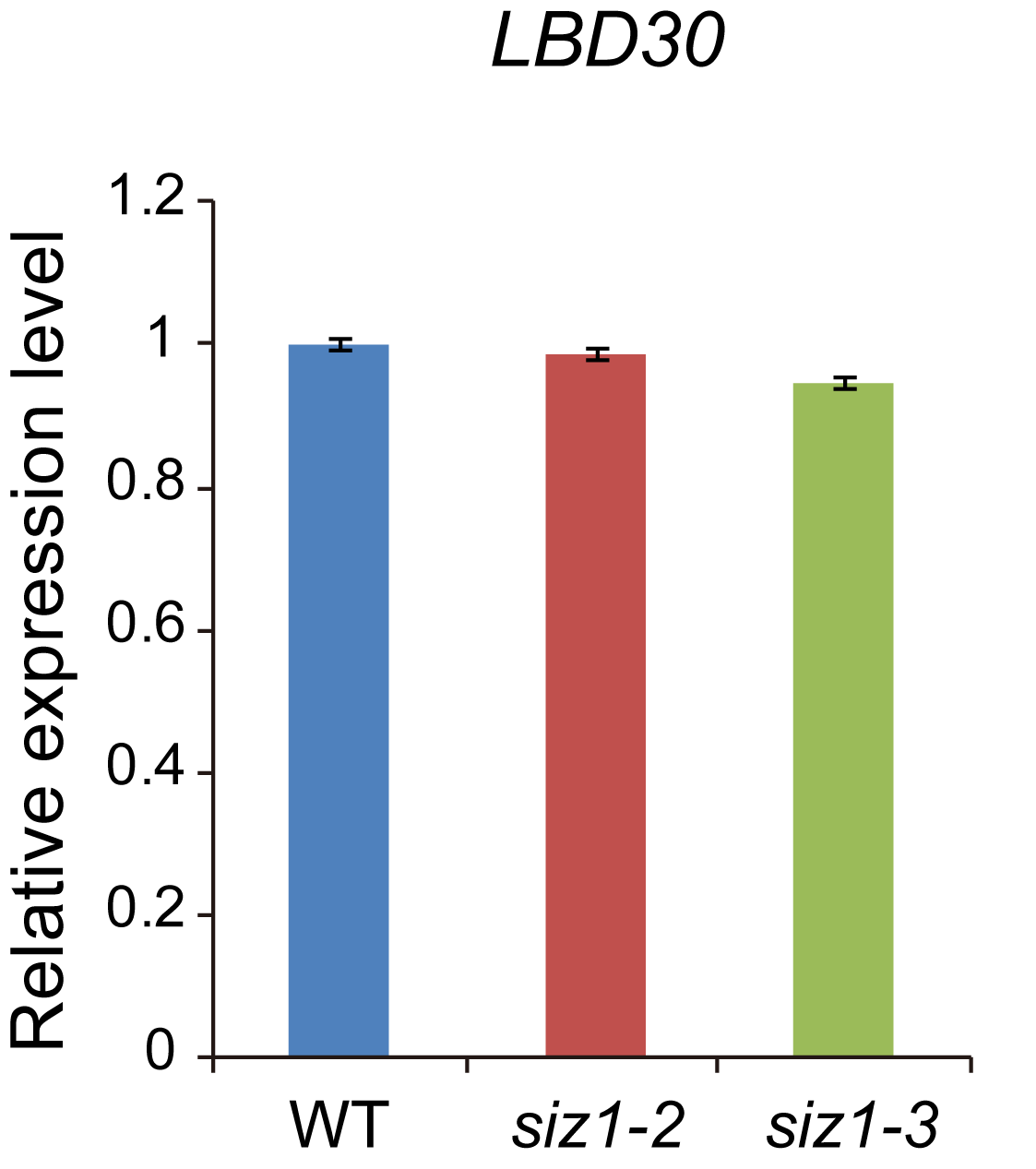

Supplement: S4 Fig — Relative levels were normalized to ACT2. The transcript level of LBD30 in WT was set to 1.0. Data represent average values±SD (n = 3 replicates). (TIF) [file pgen.1007928.s004.tif]

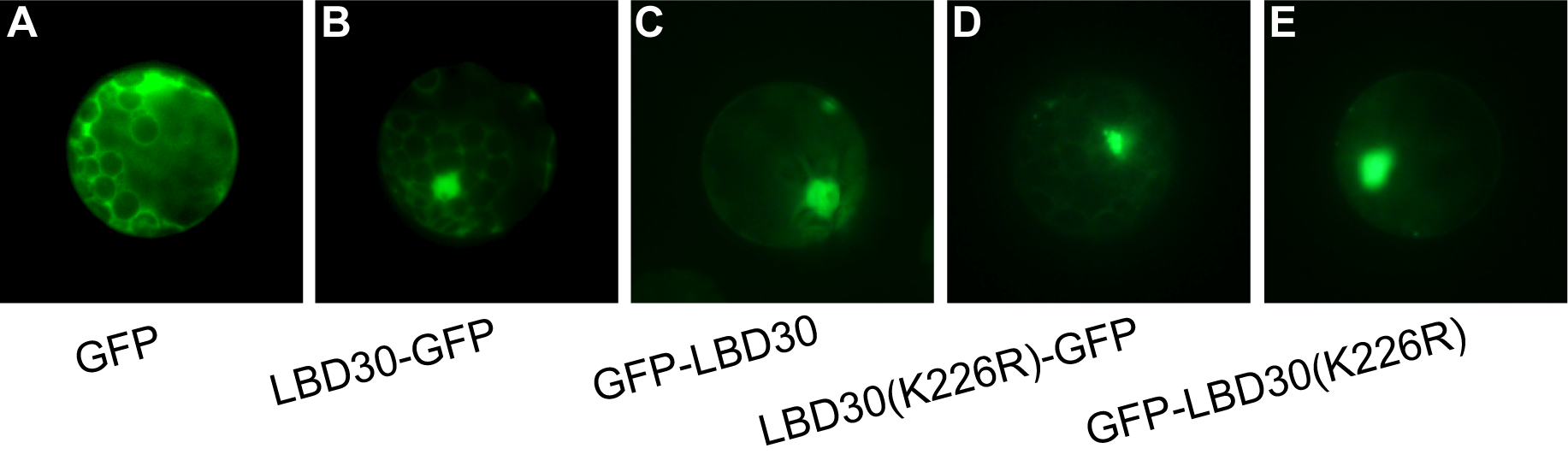

Supplement: S5 Fig — (A) A protoplast expressing GFP alone. (B, C) Protoplasts expressing GFP tagged LBD30. (D, E) Protoplasts expressing GFP tagged LBD30(K226R). (TIF) [file pgen.1007928.s005.tif]

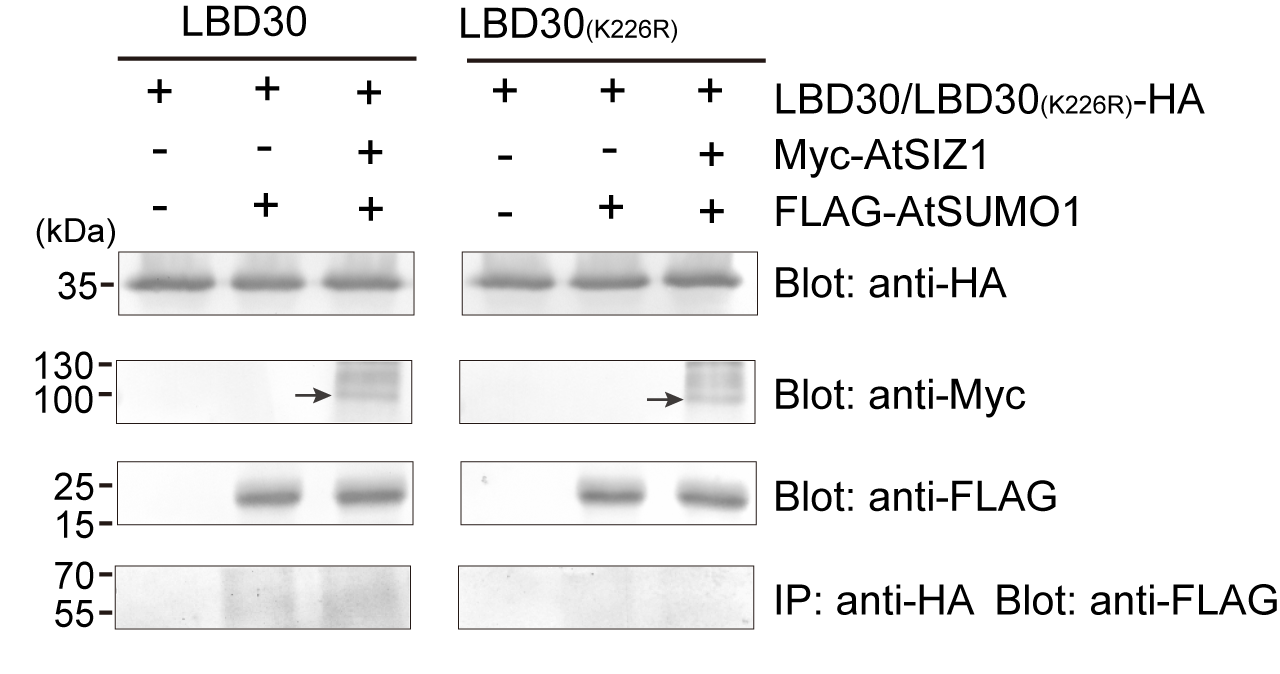

Supplement: S6 Fig — Myc tagged AtSIZ1, FLAG-tagged AtSUMO1, and HA-tagged LBD30 or LBD30(K226R) were expressed in tobacco leaves as indicated. Expression of the proteins was detected by anti-Myc, anti-HA and anti-FLAG antibodies, respectively. After immunoprecipitation with an anti-FLAG antibody, sumoylated LBD30 was detected by immunoblotting with an anti-HA antibody. Black arrows indicate Myc-AtSIZ1. (TIF) [file pgen.1007928.s006.tif]

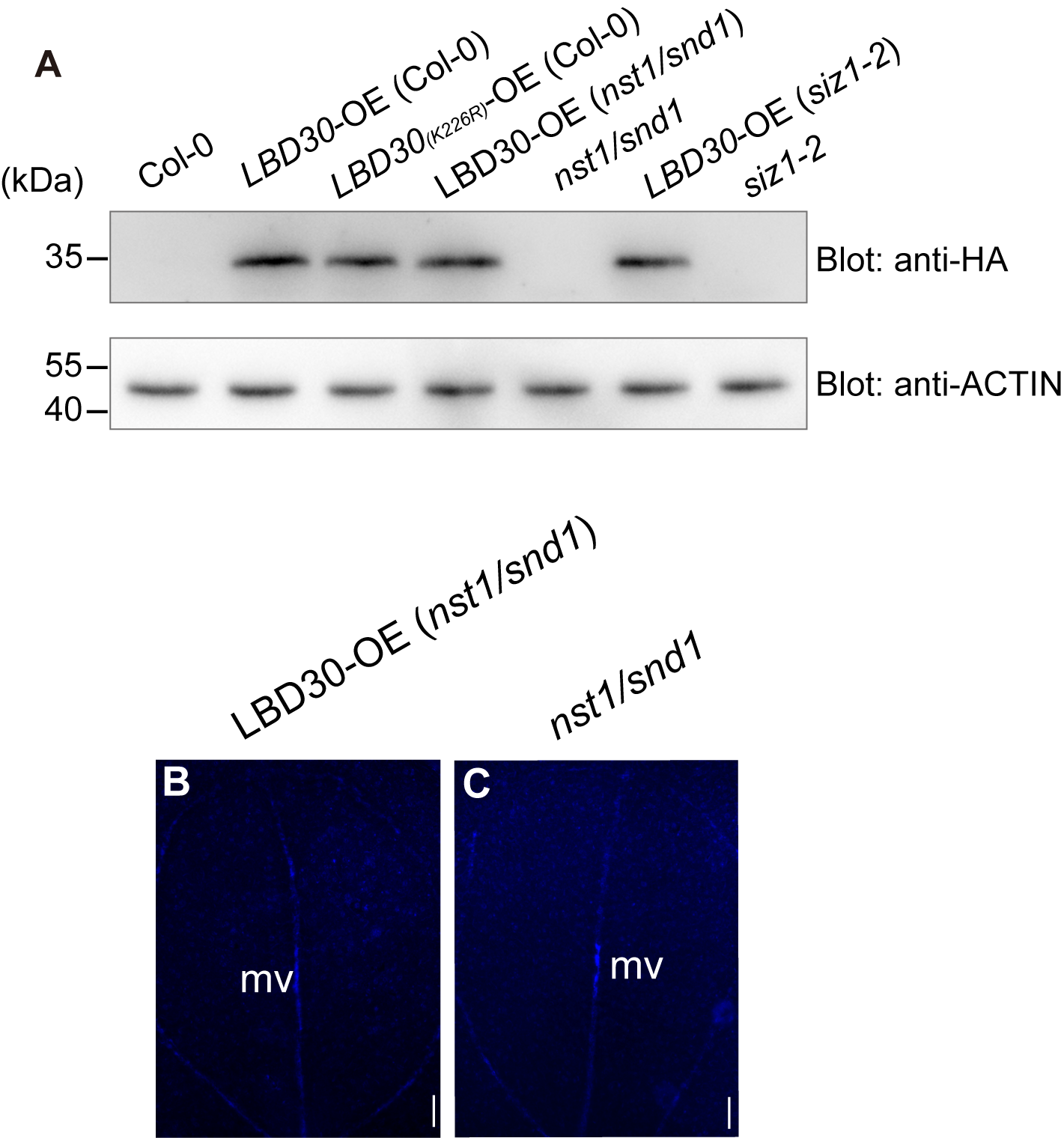

Supplement: S7 Fig — (A) The protein expression level of LBD30-cHA. ACTIN was used as an internal control, detected by an anti-ACTIN antibody (1:3000 dilution, Abmart). (B,C) Lignin autofluorescent signals of cotyledons of LBD30 overexpressing transgenic plant in the nst1/snd1 double mutant background and the nst1/snd1 double mutant (Col-0). mv, middle vein. Bars = 200 μm in (B to F). (TIF) [file pgen.1007928.s007.tif]
