# Supplementary material for: SUMO modification of LBD30 by SIZ1 regulates secondary cell wall formation in Arabidopsis thaliana
Source: PLoS Genet. 2019 Jan 18;15(1):e1007928. doi: 10.1371/journal.pgen.1007928 (PMC6355022; doi:10.1371/journal.pgen.1007928)
Supplement: S2 Table — (DOCX) [file pgen.1007928.s009.docx]

**S2 Table. Primers used in this study**.

| **Primer** | **Sequence (5‘-3’)** | **Experiment** |
| --- | --- | --- |
| SIZ1F | ATGGATTTGGAAGCTAATTGTAAG | Cloning of *AtSZI1* coding sequence |
| SIZ1R | TTAAACTCCGGTGTCTTGTCTG |  |
| LBD30F | ATGAGCAGTAGCGGAAAC | Cloning of *LBD30* and *LBD30(K226R)* |
| LBD30R | TCATTCTCGTTTTATCAC | Coding sequence |
| LBD30(K226)R | TCATTCTCGTCTTATCAC |  |
| SUMO1(GG)F | ATGTCTGCAAACCAGGAG | Cloning of *AtSUMO1* coding sequence |
| SUMO1(GG)R | TCAGCCACCAGTCTGATGGAG |  |
| PSIZ1 F | GCTGAGTGATCCCAGAGATGCACAG | Cloning of *AtSIZ1* promoter sequence |
| PSIZ1 R | GTCTTCAACACCAGACACAAACAAG |  |
| PSND1 F | GTCACATGATTGCCGTTAACAAG | Cloning of *SND1* promoter sequence |
| PSND1 R | TAACGAAGATAGCAATATATTTTTGGGTTTTTGCTC |  |
| PNST1 F | AGAACTTACTTAAAGATACGC | Cloning of *NST1* promoter sequence |
| PNST1 R | CAAAGAGATTCAAGATTAACTC |  |
| ADSIZ1 F | GTACCAGATTACGCTCATATGGATTTGGAAGCTAATTGTAAG | Yeast two hybrid assay |
| ADSIZ1 R | ATGCCCACCCGGGTGGAATTCTTAAACTCCGGTGTCTTGTCTG |  |
| BDLBD30 F | CTCAGAGGAGGACCTGCATATGAGCAGTAGCGGAAAC |  |
| BDLBD30 R | GTCGACGGATCCCCGGGAATTCTCATTCTCGTTTTATCAC |  |
| YNSIZ1 F | CGTCTATATCATGGCTCTAGACATGGATTTGGAAGCTAATTGTAAG | Bimolecular fluorescence |
| YNSIZ1 R | TCTATCGATCAATCAGGATCCTTAAACTCCGGTGTCTTGTCTG | Complementation assay |
| LBD30YC F | CACCATCACGCCATGGTCGACATGAGCAGTAGCGGAAAC |  |
| LBD30YC R | TTCTGCTTGTCCATCACTAGTACTTCTCGTTTTATCACTGACG |  |
| 28b30 F | ACAGCAAATGGGTCGGGATCCG ATGAGCAGTAGCGGAAAC | *LBD30* and *LBD30(K226R)* |
| 28b30R | GTGGTGGTGGTGGTGCTCGAG TCATTCTCGTTTTATCAC | His-tag expression construct |
| 28b30(K226R)R | GTGGTGGTGGTGGTGCTCGAG TCATTCTCGTCTTATCAC |  |
| 28bKGG F | GTGCCGCGCGGCAGCCATATGTCTGCAAACCAGGAG | AtSUMO1(KGG) |
| 28bKGG R | ACGGAGCTCGAATTCGGATCCTCAGCCACCAGTCTGATGGAG | His-tag expression construct |
| pGEXSIZ1F | GATCTGGTTCCGCGTGGATCCATGGATTTGGAAGCTAATTGTAAG | AtSIZ1 GST-tag |
| pGEXSIZ1R | CTCGAGTCGACCCGGGAATTCTTAAACTCCGGTGTCTTGTCTGATG | Expression construct |
| LBD30OE F | CATTTACGAACGATACTGCAGATGAGCAGTAGCGGAAAC | *LBD30* and *LBD30(K226R)* |
| LBD30OE R | ATCGTATGGGTACATGGTACCTCATTCTCGTTTTATCACTGACGAG | Plant overexpression construct |
| LBD30(K226R)OER | ATCGTATGGGTACATGGTACCTCATTCTCGTCTTATCACTGACGAG |  |
| SIZ1OE F | AATTTCTAGAATGGATTTGGAAGCTAATTGTAAG | *AtSIZ1* plant |
| SIZ1OE R | AATTGGTACCTTAAACTCCGGTGTCTTGTCTG | Overexpression construct |
| SUMO1F | AATTTCTAGAATGTCTGCAAACCAGGAG | *AtSUMO1* plant |
| SUMO1 R | AATTGGTACCTCAGCCACCAGTCTGATGGAG | Overexpression construct |
| 30GFP F | CATTTACGAACGATACTCGAGATGAGCAGTAGCGGAAAC | *LBD30* and *LBD30(K226R)* |
| 30GFP R | CACCATCACTAGTACGTCGACTTCTCGTTTTATCACTGACG | GFP vector construct |
| 30(K226R) GFP R | CACCATCACTAGTACGTCGACTTCTCGTcTTATCACTGACG |  |
| GFP30 F | CGAGCTGTACAAGGGTCTAGAcATGAGCAGTAGCGGAAAC |  |
| GFP30 R | TCTATCGATCAATCAGGATCCTCATTCTCGTTTTATCAC |  |
| GFP30(K226R) R | TCTATCGATCAATCAGGATCCTCATTCTCGTcTTATCAC |  |
| PSND1-LUC F | CTATAGGGCGAATTGGGTACCGTCACATGATTGCCGTTAACAAG | Dual luciferase assay |
| PSND1-LUC R | TGTTTTTGGCGTCTTCCATGGTAACGAAGATAGCAATATATTTTTGGGTTTTTGCTC |  |
| PNST1-LUC F | CTATAGGGCGAATTGGGTACCAGAACTTACTTAAAGATACGC |  |
| PNST1-LUC R | TGTTTTTGGCGTCTTCCATGGCAAAGAGATTCAAGATTAACTC |  |
| 35S30F | CATTTACGAACGATACTCGAGATGAGCAGTAGCGGAAAC |  |
| 35S30R | TCTATCGATCAATCAGGATCCTCATTCTCGTTTTATCAC |  |
| 35S30(K226R) R | TCTATCGATCAATCAGGATCCTCATTCTCGTCTTATCAC |  |
| PSIZ1GUS F | TCCTCTAGAGTCGACCTGCAG GCTGAGTGATCCCAGAGATGCACAG | *AtSIZ1* promoter-GUS construct |
| PSIZ1GUS R | TACCCTCAGATCTACCATGG GTCTTCAACACCAGACACAAACAAG |  |
| siSIZ1 1 senseF | AATTTCTAGACAGCATGTTGGCTGTGTTATTCTC |  |
| siSIZ1 1 senseR | AATTGGATCCCAAGATGTAATAATGGGTCCATC |  |
| siSIZ1 1 antiF | AATTGAGCTCCAGCATGTTGGCTGTGTTATTCTC |  |
| siSIZ1 1 antiR | AATTGGTACCCAAGATGTAATAATGGGTCCATC |  |
| siSIZ1 2 senseF | AATTTCTAGAGTTACCGGTTAAGCAAGAAGG |  |
| siSIZ1 2 senseR | AATTGGATCCCATTCATGGAAATGGAATCAAGTTC |  |
| siSIZ1 2 antiF | AATTGAGCTCGTTACCGGTTAAGCAAGAAGG |  |
| siSIZ1 2 antiR | AATTGGTACCCATTCATGGAAATGGAATCAAGTTC |  |
| qSND1 F | ACAGTCGCTGGATTCTGGAAAGC | qPCR |
| qSND1 R | TCCAATTCTCCGGACACAACTGC |  |
| qNST1 F | AGGATGTCACCGTTCATGAGGTC |  |
| qNST1 R | ACACCACCCATCCTTCGTCTTG |  |
| qMYB46 F | ACTATCTTCGTCCTGACCTCAAGC |  |
| qMYB46 R | TGCTGCAATCTGAGACCACCTG |  |
| qMYB103 F | ATGGAGTTGTGGGAAACAGGTG |  |
| qMYB103 R | GTTACTGACGGTTGATGACGACTG |  |
| qCESA4 F | AGATGCGGAGTGGAAAGAACGTG |  |
| qCESA4 R | GGTTGTCTTGCTTCAGCATCTAGG |  |
| qCESA7 F | TAGATCGGCTTTCCCTCAGGTACG |  |
| qCESA7 R | ACAGCACAGTGTTGGATGTGACG |  |
| qCESA8 F | CCGGCAAATTTATCATCCCAACGC |  |
| qCESA8 R | TTCGATACTGACTCCGCTCCATCG |  |
| qPAL1 F | GCAGTGCTACCGAAAGAAGTGG |  |
| qPAL1 R | TGTTCGGGATAGCCGATGTTCC |  |
| qCCoAOMT F | CGCCAAGAACACAATGGAGATCGG | qPCR |
| qCCoAOMT R | ACGCCGGCTTTCTCAATGATCG |  |
| q4CL1 F | TGTGGTGAAATCGAAGGATTCGG |  |
| q4CL1 R | CAACCTGTTTCGACACGAATTGC |  |
| qIRX8 F | CCGATTCGTCTTTGTTCTCTCCAC |  |
| qIRX8 R | CAAGCAAGCAAGAGAGGAGCAAG |  |
| qIRX9 F | ACTTTGGGACCCTGAGAGATGG |  |
| qIRX9 R | ATCGAATCCTGTTTGGTGCCTTC |  |
| qIRX14 F | GGACTTAGGACCATTCATGTTGGG |  |
| qIRX14 R | AGCTTCTCTTCCCTCACAACTCTC |  |
| qLBD30 F | AGACGCCGTCGTTTCAATCTGC |  |
| qLBD30 R | TGCAAACTTACCACCTGTTGCTG |  |
| qSIZ1F | GCCATCAGATGTCTACGAGGGAAG |  |
| qSIZ1R | AGAAGCAACGACGCAGTCTC |  |
| qACT2 F | TCTTCCGCTCTTTCTTTCCAAGC |  |
| qACT2 R | ACCATTGTCACACACGATTGGTTG |  |
